# Supplementary material for: Rational Design of an Ion-Imprinted Polymer for Aqueous Methylmercury Sorption
Source: Nanomaterials (Basel). 2020 Dec 17;10(12):2541. doi: 10.3390/nano10122541 (PMC7766906; doi:10.3390/nano10122541)
Supplement: Supplementary file 1 [file nanomaterials-10-02541-s001.pdf]

nanomaterials

Supporting Information

Rational design of an ion imprinted polymer for aqueous methylmercury sorption

Ruddy L. Mesa [1], Javier E.L. Villa [2,3], Sabir Khan [1,2,3], Rafaella R. Alves Peixoto [4], Marcelo A. Morgano [5], Luís Moreira Gonçalves [6], Maria D.P.T. Sotomayor [2,3,*] and Gino Picasso [1,*]

[1] Laboratory of Physical Chemistry Research, Faculty of Sciences, National University of Engineering, Lima 15333, Peru; a20156475@pucp.edu.pe (R.L.M.M.); sabir_chemist@yahoo.com (S.K.)
[2] Institute of Chemistry, State University of São Paulo (UNESP), Araraquara-SP 14800-060, Brazil; villa.javier03@gmail.com
[3] National Institute for Alternative Technologies of Detection, Toxicological Evaluation and Removal of Micropollutants and Radioactives (INCT-DATREM), Araraquara-SP 14800-060, Brazil
[4] Department of Analytical Chemistry, Fluminense Federal University (UFF), Niterói-RJ 24020-150, Brazil; rafaellapeixoto@id.uff.br
[5] Institute of Food Technology (ITAL), Campinas-SP 13070-178, Brazil; morgano.ital@gmail.com
[6] Institute of Chemistry, University of São Paulo (USP), São Paulo-SP 05508-000, Brazil; lmgoncalves@iq.usp.br
* Correspondence: m.sotomayor@unesp.br (M.D.P.T.S.); gpicasso@uni.edu.pe (G.P.); Tel.: +55-16-33019620 (M.D.P.T.S.)

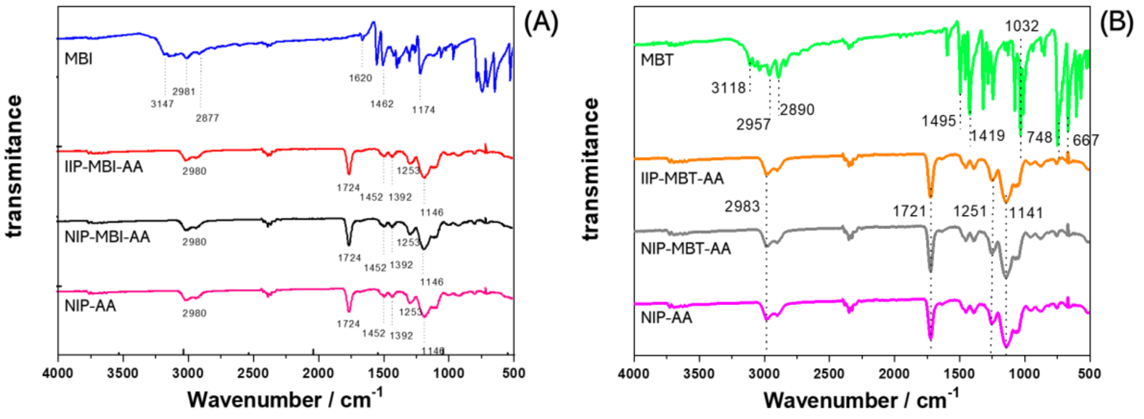

Figure S1. FTIR spectra of (A) MBI, IIP-MBI-AA, NIP-MBI-AA, and NIP-AA; and (B) MBT, IIP-MBT-AA, NIP-MBT-AA, and NIP-AA.

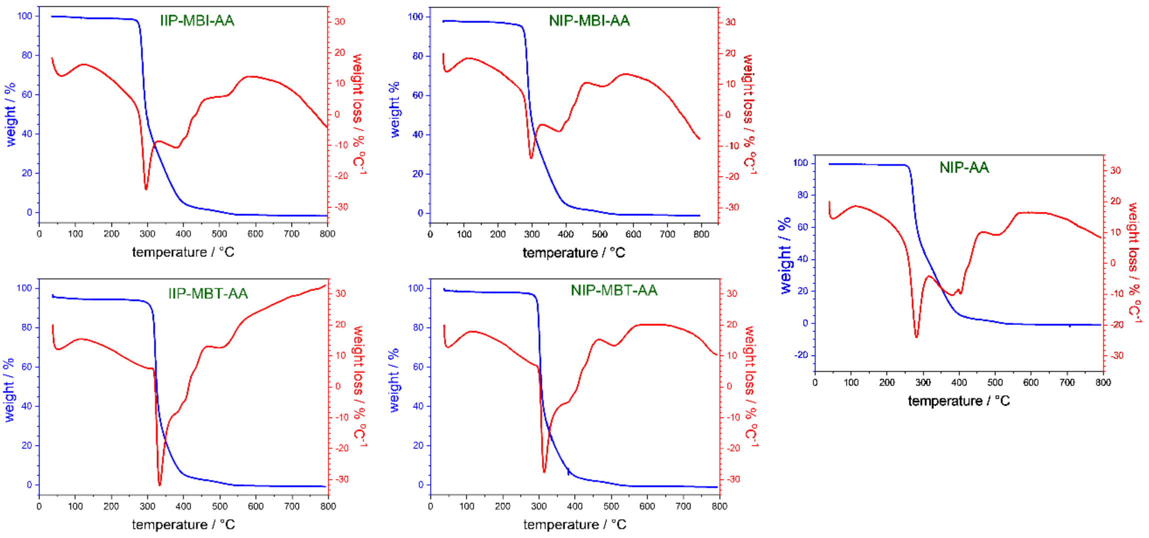

Figure S2. Thermal gravimetric (blue) and differential thermal analysis (red) of IIP-MBI-AA, NIP-MBI-AA, IIP-MBT-A, NIP-MBT-AA and NIP-AA.

Table S1. Comparative selected bond lengths (Å) for $MeHg^+$-MBI and $MeHg^+$-MBT.

material	bond´s lengths	theoretical values	experimental values
$MeHg^+$-MBI	Hg(20)-C(1)	2.232	-
	Hg(20)-S(2)	2.698	-
	S(2)-C(10)	1.739	-
	C(10)-NH(11)	1.375	-
	C(10)-N(9)	1.318	-
$MeHg^+$-MBT	Hg(11)-C(12)	2.223	2.062
	Hg(11)-S(10)	2.717	2.369
	S(10)-C(8)	1.736	1.713
	C(8)-S(9)	1.784	1.782
	C(8)-N(7)	1.297	1.323

The theoretical calculations were performed by DFT M062X and the experimental analysis for MeHg+-MBT was performed by single-crystal X-ray diffraction (Bravo *et al.* 1985).

Table S2. Predicted binding energy of $MeHg^+$-MBI and $MeHg^+$-MBT with FMs.

pre-polymerization	ΔE with FM / kcal mol^{-1}	ΔE with template $MeHg^+$ / kcal mol^{-1}
$MeHg^+$-MBI-2(AM)	-15.4	-14.8
$MeHg^+$-MBI-2(AA)	-20.0	-13.3
$MeHg^+$-MBI-2(MAA)	-18.9	-12.7
$MeHg^+$-MBI-1(4vp)	-5.9	-13.4
$MeHg^+$-MBI-1(1vim)	-5.9	-14.2
$MeHg^+$-MBI-2(2HM)	-14.9	-10.4
$MeHg^+$-MBT-1(AM)	-5.4	-9.9
$MeHg^+$-MBT-1(AA)	-12.2	-11.0
$MeHg^+$-MBT-(MAA)	-12.1	-11.2
$MeHg^+$-MBT-1(2HM)	-9.8	-9.3

AM: acrylamide, AA: acrylic acid, MAA: methacrylic acid, 4vp: 4-vinylpyridine, 1vim: 1-vinylimidazole, 2HM: 2-hydroxyethyl methacrylate

Table S3. Selectivity parameters for the adsorption of Hg^{2+}, Zn^{2+}, Cd^{2+} and Pb^{2+} ions with respect to $MeHg^{+}$.

material	ion	K_d ($MeHg^{+}$)	K_d (ion)	*k*	*I*
IIP-MBI-AA	Hg^{2+}	0.98	1.11	0.89	2.5
NIP-MBI-AA		0.35	1.01	0.35	
IIP-MBT-AA		4.38	5.07	0.86	1.0
NIP-MBT-AA		3.48	3.89	0.89	
IIP-MBI-AA	Cd^{2+}	0.74	0.04	19.8	3.6
NIP-MBI-AA		0.47	0.08	5.5	
IIP-MBT-AA		10.15	0.04	261	1.1
NIP-MBT-AA		7.5	0.03	230	
IIP-MBI-AA	Pb^{2+}	0.72	0.12	5.8	1.2
NIP-MBI-AA		0.50	0.10	5.0	
IIP-MBT-AA		7.85	0.03	288	1.0
NIP-MBT-AA		6.21	0.02	278	
IIP-MBI-AA	Zn^{2+}	0.76	0.02	35.2	2.8
NIP-MBI-AA		0.42	0.03	12.4	
IIP-MBT-AA		19.26	0.01	1510	1.3
NIP-MBT-AA		8.79	0.01	1164	

© 2020 by the authors. Submitted for possible open access publication under the terms and conditions of the Creative Commons Attribution (CC BY) license (http://creativecommons.org/licenses/by/4.0/).
